# Energy and protein intake in the Colombian population: results of the 2015 ENSIN population survey

**DOI:** 10.1017/jns.2021.2

**Published:** 2021-02-18

**Authors:** Oscar F. Herrán, Edna M. Gamboa-Delgado, María Del Pilar Zea

**Affiliations:** 1 Universidad Industrial de Santander, Carrera 32 No. 29-31, 680002, Bucaramanga, Santander, Colombia; 2 Universidad Javeriana de Cali, Facultad de Ciencias de la Salud, Cali, Valle, Colombia

**Keywords:** Nutritional surveys, Dietary proteins, Energy intake, Diet, Body weight, Colombia

## Abstract

The present study was aimed at (1) the differences between current weight *v.* ideal weight, (2) total energy intake and comparing it with required energy (Rkeer), (3) absolute protein intake in g/kg per d and g/1000 calories, (4) how energy and protein intake relate to the nutritional status of the subjects in terms of overall overweight (OEW) [overweight + obesity] and conservative overweight (CEW) [obesity] and (5) the contribution (%) of protein to total energy intake based on the acceptable macronutrient distribution range (AMDR). A dietary study was carried out in Colombia with 29 259 subjects between 1 and 64 years of age, based on cross-sectional data collected in 2015 by a 24-h dietary recall (24HR) administered as part of the National Nutrition Survey. Energy and protein intake did not differ by nutritional status. In the general population, energy intake was 2117 kcal/d (95 % CI 1969, 2264). The total protein intake was 64⋅3 g/d (95 % CI 61⋅4, 67⋅3). Adequate energy intake ranged from 90 to 100 %, except for the 1–4-year-old group, which ranged from 144 to 155 %. Protein intake was 1⋅64 g/kg per d (95 % CI 1⋅53, 1⋅75). The mean AMDR for protein to total energy intake was 13⋅3 % (95 % CI 12⋅9, 13⋅7). Excess weight began during the first 4 years of age. In conclusion, it is worth reviewing and updating energy and protein intake recommendations and dietary guidelines for the Colombian population and designing and modifying public policy.

## Introduction

In Colombia, South America, demographic, economic and epidemiological transitions have been accompanied by two other simultaneous transitions^(1)^: (1) nutritional, where overweight is more prevalent than obesity^(2)^, and (2) alimentary^(3)^, where the traditional dietary pattern may be a protective factor against developing overweight and obesity, which are mediators of cardiovascular disease and cancer^(3–5)^.

Given the limited or non-existent scientific evidence at the local level in Colombia, public policies on food insecurity, nutritional deficiencies and nutritional and dietary status have followed approaches used in other cultural and socio-economic contexts. In the general population, individual dietary practices stem from a combination of various aspects that involve the context in which people live, such as personal or individual factors, as well as external factors in the food environment, including physical, economic, sociocultural and political^(6)^. The explanations of these practices have lacked epidemiological thought, for example, multicausality.

Overweight in Colombian adults rose from 45⋅9 % in 2005 to 51⋅2 % in 2010 and 56⋅5 % in 2015, while obesity increased from 13⋅7 to 16⋅5 % and 18⋅7 % over the same period^(7)^. Nevertheless, adherence to physical activity recommendations also increased, from 19⋅9 % in 2010 to 23⋅5 % in 2015^(7)^, while the frequency of snack consumption decreased 9⋅9 % for children and 13⋅3 % for adults between 2010 and 2015^(8)^.

Food and nutrition policies impact the environment, the economy and other public policies; one example is a recommendation to update the Dietary Guidelines for Americans^(9)^. Both the amount and quality of protein consumption have been of interest due to their association with serum iron levels, cholesterol, saturated fat, the development of chronic illness, and more recently, with the carbon footprint. The impact that diet has on different environments (economic, social, cultural, industrial and technological, among others) is a complex issue and a new field of formal research. The United States Department of Agriculture proposes six categories that have an environmental impact and political importance: (1) global warming potential, (2) land use, (3) water depletion, (4) marine eutrophication, (5) availability of potable water and (6) respiratory particulate or organic material^(9)^.

In terms of the Americas, 24-h dietary recall questionnaires (24HR) have been used to estimate dietary intake in Canada (2004), Colombia (2005, 2015), Argentina (2005), Peru (2006), Mexico (2006, 2012) and the United States (1999–2016). While no significant changes in energy and protein intake were found between survey years in Mexico^(10,11)^, an increase was found in the U.S^(12)^.

In Colombia, the 2005 ENSIN^(13)^ and the Energy and Nutrients Intake Recommendations (RIEN in Spanish)^(14)^ indicate a deficiency of 36–50⋅1 % in usual protein intake. Colombia updated the RIEN in 2016 based on the epidemiological and nutritional profile of the Colombia population. This guideline sets an acceptable macronutrient distribution range (AMDR) of 10–20 % for protein for the different population groups and suggests a minimum of 15 % in order to meet the average estimated micronutrient requirement^(14)^.

The present study was aimed at determining (1) the differences between current weight *v.* ideal weight, (2) total energy intake and comparing it with required energy (Rkeer), (3) absolute protein intake in g/kg per d and g/1000 calories, (4) how energy and protein intake relate to the nutritional status of the subjects in terms of overall overweight (OEW) [overweight + obesity] and conservative overweight (CEW) [obesity] and (5) the contribution (%) of protein to total energy intake based on the AMDR.

## Materials and methods

Colombia (South America) is a middle-income country with large socio-economic inequalities. Three national nutrition surveys (ENSIN in Spanish [Encuestas Nacionales de la Situación Nutricional]) were conducted between 2005 and 2015 using a cross-sectional design. The analysis was based on data on energy (kcal/d) and protein (g/d) intake obtained from a 24HR conducted as part of the 2015 ENSIN, which was a nationally representative survey with a complex and multistage sample design. The methodological details have previously been published^(15)^.

### Population and sample

The 2015 ENSIN surveyed 44 202 urban and rural households and interviewed 151 343 subjects. The sample included 4739 segments located in 295 municipalities in the country's 32 departments and in Bogota, Colombia's capital. Various probabilistic subsamples were calculated to study specific topics, including nutrient intake, nutritional practices of interest and breast-feeding, among others. The 2015 ENSIN sample was based on the Master Household Sample for Health Studies, of the National System of Health Population Studies and Surveys, developed and implemented in 2013 by the Ministry of Health and Social Protection (7). Subjects were selected with various sampling methods, including probability, cluster, stratified and multistage. The primary sampling unit was the department, the secondary was the municipalities and the tertiary was the housing of the subjects. The subsample that constituted the 24HR included 34 099 subjects between 0 and 64 years old, 4589 of whom filled out a second 24HR. Excluded from the 34 099 were children between 0 and 1 year of age (*n* 1770), pregnant women (*n* 2589) and those without data on weight, height or both (*n* 481). The 24HR response rate was 84 %. The final sample that was analysed included 29 259 subjects.

### Source of data

Trained personnel administered the questionnaire to heads of household to obtain information on sociodemographics, food security and household wealth. Moreover, nutritionists administered the 24HR using the Automated Multiple-Pass Method (AMPM) developed in 1999 by the United States Department of Agriculture (USDA)^(15,16)^. For children under 12 years old, consumption information was supplied by the person who was responsible for having prepared and served their food the previous day and/or who accompanied the child while eating. For adolescents between 12 and 14 years old and adults 60 years or older, a rapid memory test was conducted before administering the 24HR, which included 4 of the 10 items proposed by Hodkinson in 1972 to identify dementia in elderly patients^(17)^. When one of the four questions was not adequately answered by the subject, the information was provided by the caregiver or the person responsible for feeding the child. Anthropometric measurements were taken by trained interviewers using standardised techniques and calibrated equipment. Size was determined with stadiometers (Shorr Productions LCC, Olney, MD, USA) to the nearest millimetre. SECA scales (model 874) were used to determine weight to the nearest 100 g.

The main outcome variables were OEW, CEW and energy (kcal/d) and protein (g/d) intake. Eleven covariables were also studied: sex, age, weight circumference, adherence to weekly physical activity goals, number of household members, household food security, wealth index, educational level of the head of household, ethnicity, degree of urbanism and geographic region.

### Nutritional status

Values for weight, size and sex were converted to *Z* scores based on growth references by the World Health Organization (WHO)^(17,18)^. For children up to 17 years old, OEW was based on body mass index for age (BMI/A) and *Z*-score >1, and CEW was based on *Z*-score >2. For adults, overweight was defined as ≥25 BMI <30 (kg/mt^2^), obesity as BMI ≥30, OEW = overweight + obesity and CEW = obesity. In addition, for each individual with low or excess weight, the 2015 ENSIN estimated ‘adequate weight’ based on its own data using two predictive equations (linear regressions): one for subjects between 1 and 17 years old and another for those between 18 and 64 years old. The equations included data on the sex, weight, size and age of the individuals with normal nutritional status for the age group^(19)^.

### Energy and protein intake

After rigorous quality control, the 24HR were converted to nutrients based on a food composition database specifically designed for the 2015 ENSIN. This database contains 2703 items and 9 nutritional variables. In addition, following recommendations by the 2001 FAO/WHO/UNU Expert Consultation^(20)^, the Estimated Energy Requirement (EER) was calculated for each individual based on RIEN^(14)^. Energy (kcal/d) and protein (g/d) intake were reported in absolute terms based on the first 24HR. Protein intake was reported as nutrient density in grams per 1000 calories (g/1000) and as the ratio of absolute protein intake/day (g/d) to adequate weight (Rprotein). Lastly, the ratio of absolute kcal intake/day to EER was calculated and expressed as requirement units (Rkeer) or adequacy (%EER).

### Adherence to weekly physical activity goals

The 2015 ENSIN calculated this potential confounder based on minimum adherence per week, using different methods for different age groups^(15)^. For children under 6 years old, adherence was defined as 180 min or more of play per day over the prior 7 d, according to C-MAFYCS^(21,22)^. For adolescents between 6 and 17 years old, adherence to physical activity recommendations was defined as 60 or more min/d of moderate or vigorous physical activity, according to the Youth Risk Behavior Surveillance System^(23)^. Adherence for adults was defined as at least 150 min of moderate or 75 min of vigorous aerobic physical activity/week, according to the International Physical Activity Questionnaire (IPAQ) developed by the WHO^(24)^.

Large waist circumference was defined as ≥90 cm for men and ≥80 cm for women. The degree of urbanism was categorised as urban and rural based on the population density reported by the 2015 ENSIN^(15)^. Household food security was determined with the Latin American and Caribbean Food Security Scale (ELCSA in Spanish)^(25)^. Wealth level was designated based on the index designed for the international demographics and health survey^(26)^, with the highest values representing the wealthiest subjects.

### Statistical analysis

All analyses were performed using the routines for complex sampling designs in Stata software version 14.1^(27)^. The statistical analysis was aimed at (1) describing socio-demographic characteristics, (2) for each category of covariates, describing the energy and protein intake of the subjects with prevalences (%) or averages, and presenting each one with its standard error (se) or 95 % confidence interval (95 % CI), (3) determining the prevalence ratio of protein intake to adequate weight (g/kg per d) in subjects with and without excess weight and (4) determining the average prevalence ratio of energy intake to energy required in subjects with and without excess weight.

Multiple linear regressions were performed to obtain adjusted differences in protein and energy intake between subjects with and without excess weight, and with and without conservative excess weight, with their different expressions for each category of covariates. To this end, a new term was created with the result of the cross product of intake and each category of covariates (interaction). The adjusted differences and their respective 95 % CIs incorporated the complex sample design, and the multiple regression model included the covariates sex, age, adherence to physical activity, household size, household food insecurity, wealth index, ethnicity, education level of head of household, geographic area and region.

### Ethics approval and consent to participate

All analyses were performed in accordance with the principles of the Helsinki Declaration^(28)^. The databases used are available to the public. This research is classified as ‘without risk’ according to Resolution 8430 of the Colombian Ministry of Health (1993)^(29)^. Since this is a secondary analysis of population studies with anonymised data, no authorisation was required from the Health Research Ethics Committee of the Industrial University of Santander.

## Results

A total of 29 259 subjects were studied, 48⋅8 % of which were men. The OEW of the population was 28⋅7 %: 27⋅6 % for men and 30⋅0 % for women (*P* = 0⋅123). Nine percent (9⋅0 %) of the population had CEW: 8⋅7 % of men and 9⋅2 % of women (*P* = 0⋅506). A total of 25⋅8 % of subjects met the physical activity recommendations, 37⋅5 % of households were food secure and 77⋅3 % of subjects lived in urban regions. Table 1 presents the remaining socio-demographic characteristics of the subjects studied, according to excess weight categories.

**Table 1.** Characteristics of the population studied

Variable	Total [29 259]*		Non-overall excess weight^a^ [20 844]		Overall excess weight^b^ [8415]		Conservative overweight^c^ [2631]	
	*n*^†^	%^‡^	*n*^†^	%^‡^	*n*^†^	%^‡^	*n*^†^	%^‡^
Sex								
Male	14 302	50⋅0	10 240	50⋅7	4062	48⋅1	1264	48⋅5
Female	14 937	50⋅0	10 604	49⋅3	4353	51⋅9	1367	51⋅5
Age (years)								
1–4	6294	8⋅9	4514	8⋅8	1780	9⋅4	568	9⋅6
5–12	6175	20⋅1	4424	19⋅8	1751	20⋅8	550	21⋅6
13–17	6968	11⋅0	5026	11⋅2	1942	10⋅6	602	10⋅1
18–26	1987	13⋅6	1413	13⋅9	574	13⋅0	185	13⋅0
27–49	4945	32⋅3	3462	32⋅7	1483	31⋅1	438	27⋅8
50–64	2427	14⋅1	1677	13⋅6	750	15⋅3	247	17⋅9
Physical activity								
Deficient	17 055	74⋅1	11 229	74⋅3	5826	73⋅7	1936	75⋅6
Compliant	6002	25⋅8	3950	25⋅7	2052	26⋅3	587	24⋅4
Wealth index quintile								
1 – poorest	14 651	37⋅4	10 586	37⋅3	4065	37⋅6	1303	38⋅5
2	7106	23⋅5	4989	23⋅6	2117	23⋅0	642	23⋅4
3	4834	22⋅7	3416	22⋅5	1418	23⋅4	401	18⋅7
4 – wealthiest	2668	16⋅4	1853	16⋅6	815	16⋅0	285	19⋅4
Household food security								
Food secure	9994	36⋅7	6744	34⋅9	3250	41⋅1	1051	40⋅9
Mild food insecurity	10 435	35⋅8	7455	36⋅2	2980	34⋅9	981	37⋅0
Moderate food insecurity	5317	16⋅8	3891	17⋅5	1426	15⋅0	374	13⋅2
Severe food insecurity	3509	10⋅7	2751	11⋅4	759	9⋅0	225	8⋅9
Area								
Urban	29 917	77⋅3	15 494	76⋅7	6423	78⋅7	2037	77⋅4
Rural^§^	7342	22⋅7	5350	23⋅3	1992	21⋅3	594	22⋅6

a *Non-overall excess weight:* In children and adolescents based on *Z* ≤ +1, in adults <25 (kg/m^2^).

b *Overall excess weight:* In children and adolescents based on *Z* > +1, in adults ≥25 (kg/m^2^).

c *Conservative overweight:* In children and adolescents based on *Z* > +2, in adults ≥30 (kg/m^2^).

* [*N*].

† *n* may be less than [*N*] due to missing values.

‡ Since percentages have incorporated the complex design of the sample, they may not coincide with those calculated based on absolute values.

§ The rural category included suburban population centres close to small cities, towns in rural areas distant from small towns and places dispersed or very distant from rural towns.

### Adequate and current weight

**Fig. 1.**
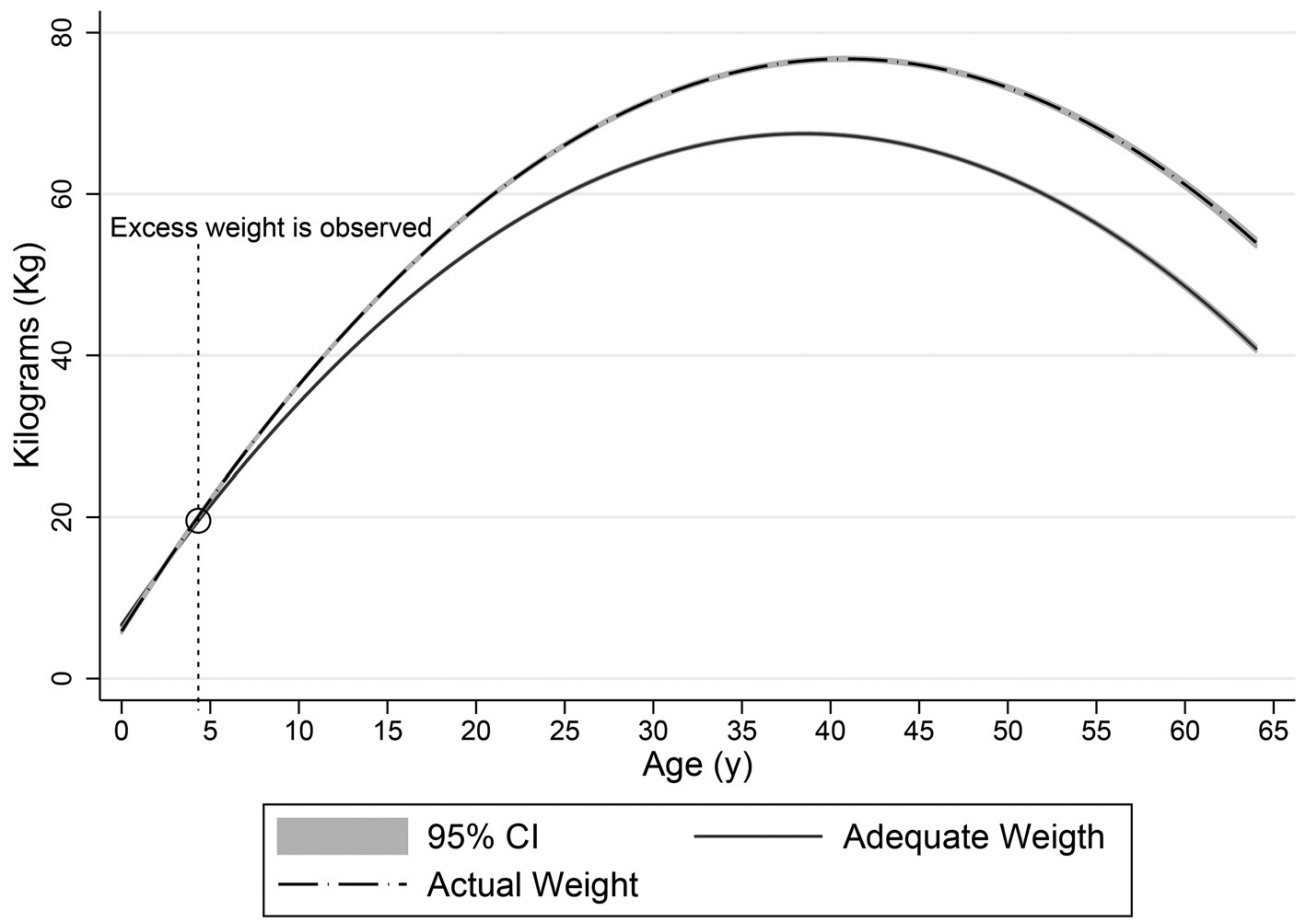
Adequate weight and current weight according to age (Colombia, 2015).

The average adequate weight was 13⋅6 kg (95 % CI 13⋅0, 14⋅2) in the 1–4-year-old group, 26 kg (95 % CI 23⋅5, 28⋅5) in the 5–12-year-old group, 50⋅6 kg (95 % CI 49⋅2, 52⋅0) in the 13–17-year-old group, 59⋅1 kg (95 % CI 58⋅4, 59⋅8) in the 18–26-year-old group, 59⋅8 kg (95 % CI 58⋅8, 60⋅7) in the 27–49-year-old group and 58⋅0 (95 % CI 56⋅7, 59⋅3) in the 50–64-year-old group (*P* < 0⋅0001). The mean differences between current and adequate weight were 0⋅5 kg (95 % CI −1⋅8, 2⋅9) in the 1–4-year-old group, 1⋅8 (−5⋅9, 9⋅6) in the 5–12-year-old group, 2⋅2 kg (95 % CI −11⋅7, 16⋅1) in the 13–17-year-old group, 4⋅5 kg (95 % CI −15⋅5, 24⋅4) in the 18–26-year-old group, 10⋅4 kg (95 % CI −14⋅6, 35⋅3) in the 27–49-year-old group and 11⋅3 kg (95 % CI −12⋅1, 34⋅7) in the 50–64-year-old group (linear trend test; *P* < 0⋅0001). Excess weight began during the first 4 years, after which the prevalence decreased in relative terms, while excess weight *v*. adequate weight remained stable (Fig. 1).

### EER and absolute energy intake

For men, EER was 2146 kcal/d (95 % CI 1985, 2308) and average intake was 2117 kcal/d (95 % CI 1969, 2264). For women, EER was 1796 kcal/d (95 % CI 1760, 1832) and intake was 1818 kcal/d (95 % CI 1740, 1896). EER and average intake by age group were as follows: EER of 1100 kcal/d (95 % CI 1075, 1126) and average intake of 1614 kcal/d (95 % CI 1320, 1908) for the 1–4-year-old age group; EER of 1533 kcal/d (95 % CI 1421, 1645) and average intake of 1787 kcal/d (95 % CI 1573, 2003) for the 5–12-year-old age group; EER of 2285 kcal/d (95 % CI 2214, 2357) and intake of 2367 kcal/d (95 % CI 2235, 2500) for the 13–17-year-old age group; EER of 2339 kcal/d (95 % CI 2295, 2382) and intake of 2305 kcal/d (95 % CI 2195, 2416) for the 18–26-year-old age group; EER of 2194 kcal/d (95 % CI 2149, 2239) and intake of 2001 kcal/d (95 % CI 1925, 2077) for the 27–49-year-old age group and EER of 2087 kcal/d (95 % CI 2050, 2125) and average intake of 1797 kcal/d (95 % CI 1731, 1862) for the 50–64-year-old age group. Supplementary Tables S1 and S2 of Supplementary material present absolute energy intake and adjusted differences for the categories of the covariables studied, according to excess weight categories.

### Relative energy consumption (ratio of (kcal/d)/EER ± se − Rkeer)

With regard to the categories of the covariables studied, children from 1 to 4 years old with CEW had the highest Rkeer (1⋅55 ± 0⋅16) and adults between 50 and 64 years old without CEW had the lowest (0⋅86 ± 0⋅83). Ethnicity was the only variable that presented statistically significant differences among subjects with excess weight. Supplementary Tables S3 and S4 of Supplementary material show these ratios according to the categories of the covariables studied.

### Protein intake in grams per 1000 kcal (g/1000)

Average protein intake was 33⋅1 g/1000 (95 % CI 31⋅9, 34⋅2) for men and 33⋅4 g/1000 (95 % CI 32⋅7, 34⋅1) for women (*P* = 0⋅668). Average protein intake was 32⋅7 g/1000 (95 % CI 31⋅8, 36⋅6) for the 1–4-year-old group, 31⋅8 g/1000 (95 % CI 30⋅8, 32⋅8) for the 5–12-year-old group, 32⋅0 g/1000 (95 % CI 30⋅2, 33⋅8) for the 13–17-year-old group, 32⋅8 g/1000 (95 % CI 32⋅0, 34⋅0) for the 18–26-year-old group, 34⋅5 g/1000 (95 % CI 32⋅8, 36⋅2) for the 27–49-year-old group and 34⋅0 g/1000 (95 % CI 32⋅6, 35⋅5) for the 50–64-year-old group (*P* = 0⋅002). No differences were found among the categories of the covariables studied when comparing protein intake (g/1000) for subjects with and without OEW. For 18–26-year olds and black subjects, statistically significant differences in protein intake (g/1000) were found when comparing subjects with and without CEW. Supplementary Tables S5 and S6 of Supplementary material present these differences.

### Protein intake in grams per kilogram of adequate weight (g/kg per d − Rprotein)

**Fig. 2.**
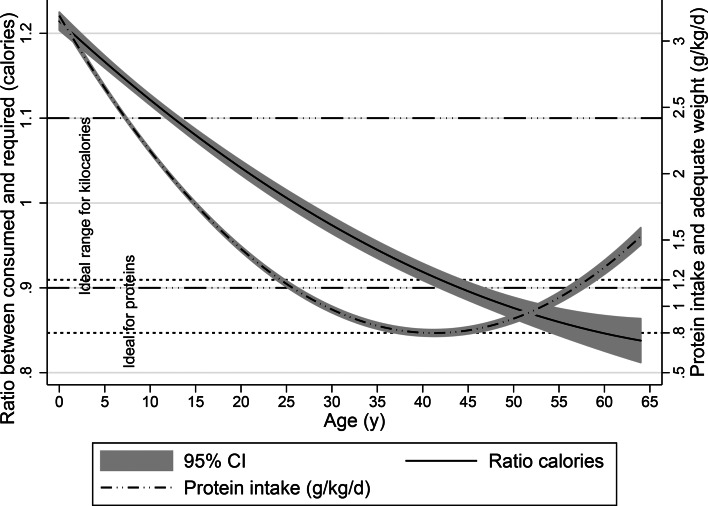
Trends in calorie and protein intake by age; the ratio between energy consumed and required; grams of protein per kilogram of adequate weight (g/kg per d) (Colombia, 2015).

Average intake was 1⋅69 g/kg per d (95 % CI 1⋅58, 1⋅79) for men and 1⋅59 g/kg per d (95 % CI 1⋅40, 1⋅78) for women (*P* = 0⋅383), 3⋅85 g/kg per d (95 % CI 3⋅46, 4⋅24) for children between 1 and 4 years of age, 2⋅25 g/kg per d (95 % CI 2⋅11, 2⋅40) for children between 5 and 12 years of age, 1⋅53 g/kg per d (95 % CI 1⋅33, 1⋅74) for adolescents between 13 and 17 years of age, 1⋅26 g/kg per d (95 % CI 1⋅21, 1⋅31) for adults between 18 and 26 years of age, 1⋅12 g/kg per d (95 % CI 1⋅08, 1⋅17) for adults between 27 and 49 years of age and 1⋅04 g/kg per d (95 % CI 0⋅98, 1⋅10) for adults between 50 and 64 years of age (*P* < 0⋅0001). Children with OEW had the highest Rprotein intake (average 3⋅94, ±0⋅25 sd). Fig. 2 summarises the findings on energy and protein intake, by age group. Tables 2 and 3 summarize the findings of protein intake (g/kg per d) regarding nutritional status.

**Table 2.** Adjusted difference in the ratio between protein intake and adequate weight (g/kg per d), between overall excess weight^a^ and non-overall excess weight^b^ for subjects in the Colombian population, 2015

Variable	Overall excess weight				Non-overall excess weight				Adjusted difference^†^	*P* Interaction
	n*	Mean	se	*P* value	n*	Mean	se	*P* value		
Sex				0⋅437				0⋅362		
Male	4062	1⋅72	0⋅06		10 240	1⋅67	0⋅05		0⋅03 (−0⋅06, 0⋅11)	0⋅554
Female	4353	1⋅61	0⋅13		10 604	1⋅58	0⋅08		0⋅03 (−0⋅10, 0⋅16)	0⋅692
Age (years)				<0⋅0001				<0⋅0001		
1–4	1780	3⋅94	0⋅25		4514	3⋅81	0⋅17		0⋅12 (−0⋅09, 0⋅33)	0⋅269
5–12	1751	2⋅24	0⋅07		4424	2⋅25	0⋅07		−0⋅01 (−0⋅10, 0⋅07)	0⋅745
13–17	1942	1⋅54	0⋅12		5026	1⋅53	0⋅10		−0⋅02 (−0⋅11, 0⋅06)	0⋅615
18–26	574	1⋅25	0⋅04		1413	1⋅27	0⋅03		−0⋅04 (−0⋅14, 0⋅05)	0⋅343
27–49	1483	1⋅12	0⋅02		1462	1⋅13	0⋅03		0⋅00 (−0⋅07, 0⋅07)	0⋅957
50–64	750	1⋅10	0⋅04		1677	1⋅03	0⋅03		0⋅03 (−0⋅06, 0⋅12)	0⋅469
Abdominal obesity^‡^				0⋅265				0⋅311		
Yes	4358	1⋅64	0⋅08		550	1⋅69	0⋅10		−0⋅03 (−0⋅18, 0⋅12)	0⋅710
No	957	1⋅72	0⋅12		3372	1⋅61	0⋅06		0⋅08 (−0⋅07, 0⋅22)	0⋅590
Physical activity				0⋅758				0⋅641		
Deficient	5826	1⋅67	0⋅09		11 229	1⋅64	0⋅05		0⋅03 (−0⋅04, 0⋅11)	0⋅360
Compliant	2052	1⋅64	0⋅06		3950	1⋅65	0⋅05		0⋅01 (−0⋅09, 0⋅10)	0⋅850
Household size				0⋅945				0⋅715		
Single person	286	1⋅63	0⋅10		237	1⋅75	0⋅12		−0⋅08 (−0⋅38, 0⋅21)	0⋅579
2–4	4919	1⋅65	0⋅06		10 227	1⋅62	0⋅04		0⋅01 (−0⋅06, 0⋅08)	0⋅785
5–6	2169	1⋅72	0⋅09		6860	1⋅67	0⋅06		0⋅06 (−0⋅04, 0⋅16)	0⋅214
7+	1041	1⋅59	0⋅14		3520	1⋅57	0⋅09		0⋅05 (−0⋅12, 0⋅23)	0⋅550
Household food security				0⋅026				0⋅082		
Food secure	3250	1⋅71	0⋅09		6744	1⋅68	0⋅05		0⋅01 (−0⋅09, 0⋅11)	0⋅874
Mild food insecurity	2980	1⋅65	0⋅06		7455	1⋅62	0⋅06		0⋅04 (−0⋅04, 0⋅12)	0⋅287
Moderate food insecurity	1426	1⋅60	0⋅11		3891	1⋅54	0⋅06		0⋅07 (−0⋅07, 0⋅21)	0⋅344
Severe food insecurity	759	1⋅58	0⋅08		2750	1⋅63	0⋅07		−0⋅06 (−0⋅19, 0⋅08)	0⋅425
Wealth index quintile				0⋅534				0⋅579		
1 – poorest	4065	1⋅74	0⋅17		10 586	1⋅66	0⋅11		0⋅07 (−0⋅06, 0⋅20)	0⋅266
2	2117	1⋅61	0⋅07		4989	1⋅65	0⋅06		−0⋅06 (−0⋅15, 0⋅03)	0⋅217
3	1418	1⋅58	0⋅07		3416	1⋅57	0⋅07		−0⋅01 (−0⋅13, 0⋅10)	0⋅805
4 – wealthiest	815	1⋅66	0⋅08		1853	1⋅61	0⋅07		0⋅08 (−0⋅08, 0⋅25)	0⋅318
Ethnicity				0⋅003				0⋅032		
Black/Afro	692	1⋅40	0⋅07		1871	1⋅47	0⋅08		−0⋅11 (−0⋅26, 0⋅04)	0⋅161
Indigenous	683	1⋅29	0⋅07		2083	1⋅46	0⋅08		−0⋅25 (−0⋅43, −0⋅08)	0⋅006
Mestizo	7011	1⋅63	0⋅08		16 827	1⋅65	0⋅06		0⋅05 (−0⋅02, 0⋅12)	0⋅140
Education of head of household				0⋅107				0⋅131		
Preschool or less	2119	1⋅54	0⋅06		6052	1⋅58	0⋅06		−0⋅02 (−0⋅10, 0⋅06)	0⋅665
Primary	2950	1⋅69	0⋅06		7062	1⋅64	0⋅05		0⋅02 (−0⋅06, 0⋅09)	0⋅659
Secondary	2750	1⋅70	0⋅11		6584	1⋅65	0⋅07		0⋅06 (−0⋅05, 0⋅17)	0⋅277
Post-secondary	563	1⋅72	0⋅10		995	1⋅70	0⋅07		−0⋅00 (−0⋅23, 0⋅22)	0⋅971
Area				0⋅015				0⋅010		
Urban	6423	1⋅71	0⋅09		15 494	1⋅68	0⋅06		0⋅04 (−0⋅05, 0⋅12)	0⋅383
Rural^§^	1992	1⋅47	0⋅04		5350	1⋅47	0⋅05		0⋅00 (−0⋅09, 0⋅09)	0⋅999
Region				0⋅948				0⋅964		
Central	2063	1⋅60	0⋅10		4956	1⋅62	0⋅08		−0⋅02 (−0⋅10, 0⋅06)	0⋅603
Atlantic	1481	1⋅84	0⋅29		4017	1⋅67	0⋅17		0⋅16 (−0⋅11, 0⋅44)	0⋅240
Oriental	1492	1⋅59	0⋅04		3672	1⋅63	0⋅04		−0⋅01 (−0⋅11, 0⋅08)	0⋅767
Pacific	1058	1⋅44	0⋅10		2813	1⋅48	0⋅09		−0⋅06 (−0⋅17, 0⋅05)	0⋅270
Bogotá	568	1⋅72	0⋅10		1485	1⋅68	0⋅09		0⋅06 (−0⋅11, 0⋅23)	0⋅484
National territories	1753	1⋅81	0⋅10		3901	1⋅76	0⋅15		0⋅04 (−0⋅15, 0⋅24)	0⋅675

a *Overall excess weight:* In children and adolescents based on *Z* > +1, in adults ≥25 (kg/m^2^).

b *Non-overall excess weight:* In children and adolescents based on *Z* ≤ +1, in adults <25 (kg/m^2^).

* Overall excess weight *n* may be less than 8415 for missing values. Non-overall excess weight *n* may be less than 20 844 for missing values.

† Adjusted difference and 95 % confidence intervals calculated with a linear regression model based on intake/day of kilocalories as the dependent variable and predictors that included indicator variables for each socio-demographic correlate, Non-overall excess weight (Overall excess weight) and cross-product (interaction) terms between overweight and indicator variables of the correlate. In addition, the linear regression model was adjusted by the following covariables: sex, age, physical activity, household size, food security, wealth index, ethnicity, education of the head of household, area and region. The complex sampling survey design was used in all multivariate regression models.

‡ In men ≥ 90 cm, in women ≥ 80 cm.

§ The rural category included suburban population centres close to small cities, towns in rural areas distant from small towns and places dispersed or very distant from rural towns.

**Table 3.** Adjusted difference in the ratio between protein intake and adequate weight (g/kg per d), between conservative overweight^a^ and non-conservative overweight^b^ for subjects in the Colombian population, 2015

Variable	Conservative overweight				Non-conservative overweight				Adjusted difference^†^	*P* interaction
	n*	Mean	sd	*P* value	n*	Mean	sd	*P* value		
Sex				0⋅756				0⋅340		
Male	1264	1⋅70	0⋅07		13 038	1⋅68	0⋅05		−0⋅01 (−0⋅11, 0⋅09)	0⋅814
Female	1367	1⋅64	0⋅16		13 590	1⋅59	0⋅09		0⋅06 (−0⋅10, 0⋅22)	0⋅483
Age (years)				<0⋅0001				<0⋅0001		
1–4	568	3⋅91	0⋅30		5726	3⋅84	0⋅18		0⋅04 (−0⋅22, 0⋅31)	0⋅738
5–12	550	2⋅27	0⋅09		5625	2⋅25	0⋅07		0⋅00 (−0⋅10, 0⋅11)	0⋅979
13–17	602	1⋅45	0⋅11		6366	1⋅54	0⋅10		−0⋅09 (−0⋅20, 0⋅01)	0⋅085
18–26	185	1⋅17	0⋅06		1802	1⋅27	0⋅03		−0⋅13 (−0⋅27, 0⋅02)	0⋅079
27–49	438	1⋅10	0⋅04		4507	1⋅13	0⋅02		−0⋅02 (−0⋅11, 0⋅07)	0⋅666
50–64	247	1⋅15	0⋅07		2180	1⋅03	0⋅03		0⋅13 (−0⋅02, 0⋅29)	0⋅097
Abdominal obesity^‡^				0⋅599				0⋅618		
Yes	1808	1⋅64	0⋅09		3100	1⋅65	0⋅07		0⋅00 (−0⋅10, 0⋅11)	0⋅935
No	31	1⋅78	0⋅30		4298	1⋅63	0⋅07		0⋅06 (−0⋅42, 0⋅54)	0⋅791
Physical activity				0⋅940				0⋅878		
Deficient	1936	1⋅67	0⋅10		15 119	1⋅65	0⋅06		0⋅02 (−0⋅07, 0⋅11)	0⋅618
Compliant	587	1⋅68	0⋅10		5415	1⋅65	0⋅05		0⋅03 (−0⋅11, 0⋅17)	0⋅695
Household size				0⋅187				0⋅516		
Single person	112	1⋅54	0⋅14		411	1⋅73	0⋅09		−0⋅19 (−0⋅52, 0⋅13)	0⋅244
2–4	1603	1⋅62	0⋅08		13 543	1⋅63	0⋅05		−0⋅02 (−0⋅11, 0⋅07)	0⋅627
5–6	642	1⋅75	0⋅11		8387	1⋅68	0⋅06		0⋅07 (−0⋅07, 0⋅20)	0⋅337
7+	274	1⋅79	0⋅22		4287	1⋅56	0⋅09		0⋅26 (−0⋅05, 0⋅58)	0⋅101
Household food security				0⋅613				0⋅026		
Food secure	1051	1⋅71	0⋅12		8943	1⋅69	0⋅05		−0⋅02 (−0⋅15, 0⋅12)	0⋅791
Mild food insecurity	981	1⋅63	0⋅07		9454	1⋅63	0⋅06		0⋅02 (−0⋅09, 0⋅12)	0⋅782
Moderate food insecurity	374	1⋅61	0⋅17		4943	1⋅55	0⋅07		0⋅09 (−0⋅15, 0⋅33)	0⋅453
Severe food insecurity	225	1⋅71	0⋅12		3284	1⋅61	0⋅07		0⋅12 (−0⋅11, 0⋅35)	0⋅312
Wealth index quintile				0⋅599				0⋅551		
1 – poorest	1303	1⋅77	0⋅21		13 348	1⋅68	0⋅12		0⋅06 (−0⋅08, 0⋅20)	0⋅374
2	642	1⋅54	0⋅07		6464	1⋅65	0⋅06		−0⋅12 (−0⋅25, 0⋅00)	0⋅057
3	401	1⋅64	0⋅08		4433	1⋅57	0⋅07		0⋅04 (−0⋅11, 0⋅20)	0⋅574
4 – wealthiest	285	1⋅64	0⋅10		2383	1⋅62	0⋅07		0⋅06 (−0⋅14, 0⋅26)	0⋅551
Ethnicity				0⋅027				0⋅012		
Black/Afro	268	1⋅41	0⋅09		2295	1⋅45	0⋅07		−0⋅07 (−0⋅27, 013)	0⋅496
Indigenous	201	1⋅50	0⋅13		2565	1⋅42	0⋅07		−0⋅07 (−0⋅39, 0⋅24)	0⋅643
Mestizo	2152	1⋅70	0⋅10		21 686	1⋅66	0⋅06		0⋅04 (−0⋅06, 0⋅13)	0⋅449
Education of the head				0⋅046				0⋅116		
Preschool or less	673	1⋅50	0⋅08		7498	1⋅57	0⋅05		−0⋅05 (−0⋅19, 0⋅09)	0⋅467
Primary	894	1⋅69	0⋅07		9118	1⋅65	0⋅05		0⋅02 (−0⋅08, 0⋅13)	0⋅657
Secondary	874	1⋅72	0⋅15		8460	1⋅66	0⋅07		0⋅05 (−0⋅09, 0⋅19)	0⋅493
Post-secondary	179	1⋅85	0⋅13		1379	1⋅69	0⋅06		0⋅14 (−0⋅12, 0⋅41)	0⋅278
Area				0⋅127				0⋅007		
Urban	2037	1⋅70	0⋅11		19 880	1⋅66	0⋅06		0⋅01 (−0⋅09, 0⋅11)	0⋅865
Rural^§^	594	1⋅52	0⋅06		6748	1⋅47	0⋅04		0⋅09 (−0⋅04, 0⋅21)	0⋅172
Region				0⋅812				0⋅973		
Central	685	1⋅59	0⋅12		6334	1⋅62	0⋅09		−0⋅04 (−0⋅16, 0⋅08)	0⋅491
Atlantic	536	1⋅88	0⋅30		4962	1⋅70	0⋅19		0⋅15 (−0⋅07, 0⋅36)	0⋅171
Oriental	404	1⋅53	0⋅08		4760	1⋅63	0⋅04		−0⋅08 (−0⋅26, 0⋅09)	0⋅344
Pacific	339	1⋅45	0⋅10		3532	1⋅47	0⋅09		−0⋅02 (−0⋅18, 0⋅13)	0⋅749
Bogotá	136	1⋅86	0⋅15		1917	1⋅69	0⋅09		0⋅15 (−0⋅12, 0⋅43)	0⋅272
National territories	531	1⋅83	0⋅12		5123	1⋅77	0⋅14		0⋅05 (−0⋅21, 0⋅30)	0⋅722

a *Conservative overweight:* In children and adolescents based on *Z* > +2, in adults ≥30 (kg/m^2^).

b *Non-conservative overweight:* In children and adolescents based on *Z* ≤ +2, in adults <30 (kg/m^2^).

* Conservative overweight *n* may be less than 2631 for missing values. Non-conservative overweight *n* may be less than 26 628 for missing values.

† Adjusted difference and 95 % confidence intervals calculated with a linear regression model based on consumption/day of kilocalories as a dependent variable and predictors that include indicator variables for each socio-demographic correlate, Non-conservative overweight (Conservative overweight) and cross-product (interaction) terms between obese and indicator variables of the correlate. In addition, the linear regression model was adjusted by the following covariables: sex, age, physical activity, household size, food security, wealth index, ethnicity, education of the head, area and region. The complex sampling survey design was used in all multivariate regression models.

‡ In men ≥ 90 cm, in women ≥ 80 cm.

§ The rural category included suburban population centres close to small cities, towns in rural areas distant from small towns and places dispersed or very distant from rural towns.

### Relative contribution of protein to total energy, AMDR (%)

Proteins made up 13⋅27 % (95 % CI 12⋅88, 13⋅66) of the total energy consumed by men and 13⋅31 % (95 % CI 13⋅06, 13⋅56) of the total consumed by women (*P* = 0⋅834). The contribution of protein to total energy intake was 13⋅07 % (95 % CI 12⋅72, 13⋅43) for the 1–4-year-old age group, 12⋅71 % (95 % CI 12⋅30, 13⋅11) for the 5–12-year-old age group, 12⋅82 % (95 % CI 12⋅09, 13⋅54) for the 13–17-year-old age group, 13⋅13 % (95 % CI 12⋅78, 13⋅47) for the 18–26-year-old age group, 13⋅79 % (95 % CI 13⋅11, 14⋅46) for the 27–49-year-old age group and 13⋅61 % (95 % CI 13⋅05, 14⋅18) for the 50–64-year-old age group. Supplementary Fig. S1 of Supplementary material presents the AMDR of protein (%) to total energy intake.

## Discussion

This work found that excess weight in the Colombian population is detectable by the age of 4 years, which may suggest that the excessive energy and protein consumption described herein may be the causal pathway to excess weight and obesity at an early age. Once excess weight begins, it increases steadily until age 40, and although it decreases after age 40, there continues to be a gap between adequate and current weight (Fig. 1). With regard to current mean energy and protein intake, no significant differences were found between subjects with and without OEW and CEW, with the exception of ethnicity. In general, the Rkeer was found to fall within the suggested range of 90–110 %, except for children between 1 and 4 years old (144 and 155 %). Given the low level of physical activity on the part of the Colombian population^(7)^, if a range of 0⋅8–1⋅2 g/kg per d is considered adequate protein consumption regardless of the nutritional condition of the subjects^(30)^, then the population consumes more protein than the limit. Nevertheless, long-term protein consumption of up to 2⋅0 g/kg per d has been considered to be safe^(30)^, and protein intake has been considered inadequate when the relative protein contribution is under the RIEN^(14)^ recommendation of 15 %.

The prevalence of overweight and obesity among Colombian adults was greater for women than for men: 8 and 6⋅8 %, respectively^(7)^. Since overweight, obesity, OEW and CEW cannot be attributed to the prior day's energy or macronutrient intake, as expected, absolute and relative energy and protein intake did not differ according to the nutritional condition of these subjects. Excess weight is a long-term condition that is related only to usual intake and not to current intake based on a single 24HR^(31)^.

Proteins are one of the most commonly studied macronutrients. They are required for growth and development, and for the synthesis of essential amino acids^(30)^. The U.S. National Academy of Medicine (formerly the Institute of Medicine [IOM]) established the estimated average requirement (EAR) and recommended dietary requirement (RDA) in grams per kilograms per day (g/kg per d) (EAR of 0⋅66 g/kg per d and RDA of 0⋅80 g/kg per d)^(32)^, while RIEN established the relative contribution of protein to total energy intake, with a relative contribution between 10 and 20 % (equivalent to 45 g/d for children between 2 and 5 years of age and up to 103 g/d for subjects between 14 and 17 years old)^(14)^. In all cases studied herein, the estimates exceeded both IOM and RIEN recommendations and were similar to those reported by the 2003–2004 National Health and Nutrition Examination Survey (NHANES): average of 56 g/d ± 14 sd for children and adolescents, 91 ± 22 g/d for subjects between 19 and 30 years of age, 86 g/d ± 20 sd for adults for adults between 30 and 50 years and 66 ± 17 g/d for adults over 50 years old^(33)^. NHANES reported that between 1999 and 2016, the AMDR for total proteins (both animal and vegetable) increased from 15⋅5 to 16⋅4 % in the U.S. population over 20 years of age^(12)^. Furthermore, protein intake in 2003–6 did not differ significantly from protein intake in 2015–16^(34)^.

Excess protein intake has been associated with increased mortality from cardiovascular disease, with a hazard ratio of 1⋅08 per 10 % increase in energy consumption (95 % CI 1⋅01, 1⋅16; *P* = 0⋅04 for the trend). This was found among those who had at least one risk factor associated with smoking, heavy alcohol consumption, overweight, obesity or sedentarism^(35,36)^. In a cohort study of Swedish women between 30 and 49 years old with a 16-year follow-up, regular consumption of high-protein, low-carbohydrate diets was associated with a greater risk of cardiovascular disease, with an incidence rate of 1⋅05 (95 % CI 1⋅02, 1⋅08)^(37)^. A case–control study of the Korean population between 30 and 76 years old reported an inverse association, though not statistically significant, between vegetable protein intake and the prevalence of colorectal adenoma, after adjusting for age, total energy intake, waist circumference, BMI, HDL-cholesterol, fasting glucose, alcohol intake and smoking. Nevertheless, when adjusted by potential confounders, that association was not significant (OR 0⋅54 [95 % CI 0⋅27, 1⋅11]; *P* = 0⋅13 for the trend)^(38)^. With regard to the risk of type 2 diabetes mellitus, a cohort study of the population in China between 20 and 74 years old found that, over the long term, diets low in carbohydrates, high in fats and high in proteins were associated with a higher risk of type 2 diabetes for subjects who consumed excess calories (RR 1⋅64 [95 % CI 1⋅03, 2⋅61]; *P* = 0⋅040 for the trend)^(39)^. A cohort study with an Iranian population over 20 years old found that the risk of incidence of chronic kidney disease increased with an increase in high-protein, low-carbohydrate diets (OR 1⋅48 [95 % CI 1⋅03, 2⋅15]; *P* = 0⋅027 for the trend)^(40)^. Excess protein consumption of over 0⋅8 g/kg per d increased intraglomerular pressure and complications from chronic kidney disease^(41)^. Lastly, the EAT–Lancet Commission has presented robust evidence of the health and environmental consequences from consuming over 0⋅8 g/kg per d of protein, or ≥10 % of the AMDR, predominantly from animal sources. The commission reported health consequences such as an association with overall mortality from cancer, cardiovascular disease and type 2 diabetes, and an environmental impact on the production of greenhouse gases (methane, nitrous oxide and carbon dioxide) from food production^(42)^.

### Public policy implications

The development of the science of nutrition cannot be understood without the institutions and policies that are associated with them^(43)^. In this case, energy and protein intake is not an isolated event that occurs outside the socio-economic and cultural context of the subjects. Furthermore, public policy can provide proper guidance on multiple levels^(43)^. The evidence presented herein, based on data on protein and energy intake from the 2015 ENSIN, will help institutions that are responsible for improving diets and nutrition^(14)^ to not only review and modify nutritional goals but also sustain them, as suggested by the Dietary Guidelines for Americans^(9)^. Incorporating sustainability in a possible update of the RIEN and the Food-based Dietary Guidelines (GABAS in Spanish)^(44)^ may also improve food security in the country^(9,45)^. Future modifications could include conscious eating as promoted by Canadian dietary guidelines^(46)^ and standardising recommendations in g/kg per d^(33)^. Protein intake limits and the sources of protein can also be adapted, given that red meat and eggs can play a protective role in iron deficiency anemia, thereby reducing its prevalence in Colombia^(47)^. By having identified the age at which the current and adequate weight of the subjects began to deviate from each other, dietary and nutritional education as well as other multi-sector dietary interventions can be better targeted before excess weight develops. Various actions need to be taken to prevent pseudoscience from replacing food and nutrition education, such as compelling messages and actions regarding how long a diet needs to be followed before harm or benefits result, the complexity of interactions among different levels of risk factors and differences in individual and population interventions carried out in particular cultural contexts. Lastly, the data on the prevalence of protein intake deficiency reported in the 2005 ENSIN should be reviewed to take into account habitual or long-term consumption^(13)^.

### Strengths and limitations of the study

The primary limitation of the present study was the inability to determine causal associations due to the cross-sectional design of the ENSIN. Another limitation was that the present study reported current intake rather than usual or long-term intake, given the use of the 24HR method. Sources of protein could also not be determined (animal or vegetable). Nevertheless, this analysis also has its strengths. The data were taken from a nationally representative survey that estimated energy and protein intake based on a 24HR that was administered by well-trained nutritionists. Furthermore, the 24HR was translated into nutrients based on the best food composition database available in Colombia.

## Supplementary material

For supplementary material accompanying this paper visit https://doi.org/10.1017/jns.2021.2. click here to view supplementary material
