# Supplementary material for: Energy and protein intake in the Colombian population: results of the 2015 ENSIN population survey
Source: J Nutr Sci. 2021 Feb 18;10:e11. doi: 10.1017/jns.2021.2 (PMC8057517; doi:10.1017/jns.2021.2)
Supplement: Supplementary file 1 [file S2048679021000021sup001.pdf]

Energy and protein intake in the Colombian population: results of the 2015 ENSIN population survey

Oscar F. Herrán, Edna M. Gamboa-Delgado, María del Pilar Zea

Online Supplementary Material

Table 1S Adjusted difference in energy/day intake (kilocalories). Overall excess weight and non-overall excess weight subjects. Colombian population, 2015.

Variable	Overall excess weight*				Non-overall excess weight				Adjusted difference‡	*P* Interaction
	n†	Mean	SE	*P* value	n†	Mean	SE	*P* value		
Sex				0.032				<0.0001		
Male	4062	2078	93		10240	2131	68		-57 (-137, 22)	0.152
Female	4353	1847	51		10604	1806	37		45 (-20, 111)	0.174
Age (years)				0.419				0.083		
1-4	1780	1690	184		4514	1582	130		144 (-18, 305)	0.081
5-12	1751	1762	118		4424	1799	104		-51 (-118, 15)	0.127
13-17	1942	2367	79		5026	2367	64		67 (-85, 98)	0.885
18-26	574	2356	76		1413	2285	61		64 (-115, 242)	0.478
27-49	1483	1995	49		3462	2003	39		30 (-54, 113)	0.481
50-64	750	1745	43		1677	1820	40		-62 (-167, 43)	0.245
Abdominal obesity§				0.790				0.945		
Yes	4358	1966	44		550	1984	65		3 (-121, 127)	0.962
No	957	1987	99		3372	1989	55		14 (-132, 161)	0.845
Physical activity				0.593				0.805		
Fails	5826	1967	48		11229	1973	37		4 (-53, 61)	0.897
Complies	2052	1944	54		3950	1981	45		-28 (-121, 64)	0.541
Household size				0.376				0.534		
Unipersonal	286	2076	80		237	2057	105		21 (-249, 290)	0.879
2-4	4919	1938	45		10227	1973	41		-35 (-99, 28)	0.271
5-6	2169	1965	65		6860	1980	38		13 (-85, 110)	0.798
7+	1041	2028	71		3520	1938	48		101 (-25, 226)	0.113
Food security in the home				0.782				0.324		
Food secure	3250	1959	64		6744	1987	46		-24 (-102, 54)	0.539
Mild insecurity	2980	1952	43		7455	1969	37		-8 (-77, 62)	0.822
Moderate insecurity	1426	1939	59		3891	1977	48		-35 (-141, 71)	0.506
Severe insecurity	759	2010	64		2750	1920	50		115 (-29, 259)	0.114
Wealth index quintile				0.651				0.626		
1- poorest	4065	1913	984		10586	1926	72		-11 (-82, 60)	0.765
2	2117	1974	56		4989	2013	49		-52 (-147, 44)	0.283
3	1418	2053	55		3416	2026	53		31 (-70, 132)	0.540
4- wealthiest	815	1904	61		1853	1938	43		28 (-124, 179)	0.716
Ethnicity				0.376				0.873		
Black/Afro	692	1921	72		1871	2013	65		-120 (-256, 17)	0.085
Indigenous	683	1753	92		2083	1741	117		-86 (-303, 131)	0.432
Mestizo	7011	1969	51		16827	1977	39		9 (-47, 64)	0.752
Education of the head				0.808				0.619		
Preschool or less	2119	1938	50		6052	2017	42		-67 (-149, 15)	0.106
Primary	2950	1959	60		7062	1925	37		35 (-49, 120)	0.410
Secondary	2750	1977	56		6584	1974	46		24 (-68, 116)	0.602
Post-secondary	563	1903	71		995	2009	67		-148 (-345, 48)	0.137
Area				0.391				0.767		
Urban area	6423	1943	55		15494	1967	43		-9 (-66, 47)	0.743
Rural‖	1992	2013	60		5350	1985	45		7 (-85, 99)	0.878
Region				0.936				0.748		
Central	2063	1876	115		4956	1945	101		-71 (-158, 15)	0.104
Atlantic	1481	2079	95		4017	2051	58		25 (-109, 160)	0.708
Oriental	1492	2004	48		3672	1940	44		76 (-10, 162)	0.084
Pacific	1058	1929	77		2813	1957	72		-33 (-157, 91)	0.594
Bogotá	568	1924	67		1485	1938	69		49 (-75, 174)	0.432
National territories	1753	1856	59		3901	1985	66		-139 (-287, 10)	0.067

* **Overall excess weight** In children and adolescents based on Z> +1, in adults≥ 25 (Kg/m^2) † In Overall excess weight *n* may be less than 8415 for missing values. In Non-overall excess weight *n* may be less than 20844 for missing values. ‡ Adjusted difference and 95% confidence interval achieved in a linear regression model with the consumption/day of kilocalories as a dependent variable and predictors that include indicator variables for each sociodemographic correlates, Non-overall excess weight (Overall excess weight) and cross-product (interaction) terms between overweight and indicator variables of the correlate. In addition, the linear regression model was adjusted by the following covariables; sex, age, physical activity, household size, food security, wealth index, ethnicity, education of the head, area and region. The complex sampling survey design was taken into account in all multivariate regression models. § In men≥90 cm, in women≥80 cm. ‖ The rural category included suburban population centers close to small cities, towns in rural areas distant from small towns, and disperses or very distant from rural towns.

Table 2S Adjusted difference in energy/day intake (kilocalories). Conservative overweight and non-conservative overweight subjects. Colombian population, 2015.

Variable	Conservative overweight [*]				Non-conservative overweight				Adjusted difference‡	*P* Interaction
	n†	Mean	SE	*P* value	n†	Mean	SE	*P* value		
Sex				0.040				<0.0001		
Male	1264	2074	112		13038	2121	71		-46 (-155, 63)	0.404
Female	1367	1813	56		13590	1819	39		-8 (-85, 69)	0.833
Age (years)				0.349				0.135		
1-4	568	1725	215		5726	1602	139		-145 (-20, 311)	0.084
5-12	550	1734	122		5625	1794	106		-102 (-177, -27)	0.008
13-17	602	2239	95		6366	2379	64		-102 (-216, 11)	0.077
18-26	185	2317	124		1802	2304	58		6 (-264, 277)	0.962
27-49	438	2013	73		4507	2000	38		19 (-118, 157)	0.781
50-64	247	1787	79		2180	1798	34		33 (-132, 197)	0.694
Abdominal obesity§				0.211				0.789		
Yes	1808	1950	54		3100	1979	43		-28 (-110, 53)	0.490
No	31	1731	190		4298	1990	59		-264 (-597, 69)	0.118
Physical activity				0.195				0.819		
Fails	1936	1968	60		15119	1971	38		-0 (-81, 80)	0.994
Complies	587	1884	68		5415	1978	43		-105 (-223, 14)	0.082
Household size				0.088				0.596		
Unipersonal	112	1875	102		411	2119	79		-266 (-509, -23)	0.033
2-4	1603	1920	55		13543	1967	40		-29 (-108, 50)	0.469
5-6	642	1905	83		8387	1982	40		-83 (-219, 53)	0.229
7+	274	2166	124		4287	1943	47		197 (-36, 430)	0.096
Food security at home				0.351				0.378		
Food secure	1051	1929	73		8943	1984	48		-63 (160, 35)	0.204
Mild insecurity	981	1909	58		9454	1970	36		-49 (-142, 44)	0.295
Moderate insecurity	374	1946	98		4943	1969	47		-10 (-206, 187)	0.923
Severe insecurity	225	2103	138		3284	1928	47		201 (-81, 483)	0.159
Wealth index quintile				0.367				0.680		
1- poorest	1303	1861.7	109		13348	1929	76		-48 (-139, 42)	0.291
2	642	1964.6	77		6464	2005	48		-85 (-230, 59)	0.242
3	401	2052.8	69		4433	2032	50		18 (-117, 153)	0.794
4- wealthiest	285	1953.2	95		2383	1925	39		95 (-92, 281)	0.315
Ethnicity				0.279				0.788		
Black/Afro	268	1847	83		2295	2005	63		-178 (-339, -19)	0.029
Indigenous	201	1820	174		2565	1745	106		35 (-340, 410)	0.853
Mestizo	2152	1952	62		21686	1977	40		-14 (-87, 59)	0.699
Education of the head				0.526				0.643		
Preschool or less	673	1910	72		7498	2005	41		-77 (-207, 54)	0.244
Primary	894	1932	75		9118	1935	39		-12 (-121, 97)	0.824
Secondary	874	1950	72		8460	1977	46		-9 (-129, 111)	0.833
Post-secondary	179	1995	104		1379	1970	56		-3 (-216, 210)	0.978
Area				0.963				0.574		
Urban area	2037	1940	656		19880	196	44		-17 (-91, 57)	0.647
Rural[ǀ]	594	1935	84		6748	1997	45		-66 (-210, 78)	0.364
Region				0.990				0.778		
Central	685	1829	121		6334	1936	104		-124 (-246, -2)	0.046
Atlantic	536	2114	97		4962	2053	64		62 (-71, 195)	0.355
Oriental	404	1935	75		4760	1962	42		8 (-128, 143)	0.910
Pacific	339	1945	98		3532	1949	72		11 (-184, 206)	0.911
Bogotá	136	1881	108		1917	1938	65		-41 (-249, 168)	0697
National territories	531	1799	67		5123	1958	61		-158 (-348, 31)	0.100

[*] **Conservative overweight** In children and adolescents based on Z > +2, in adults ≥ 30 (Kg/m^2) † In conservative overweight *n* may be less than 2631 for missing values. In Non-conservative overweight *n* may be less than 26628 for missing values. ‡ Adjusted difference and 95% confidence interval achieved in a linear regression model with the consumption/day of kilocalories as a dependent variable and predictors that include indicator variables for each sociodemographic correlates, Non-conservative overweight (Conservative overweight) and cross-product (interaction) terms between obese and indicator variables of the correlate. In addition, the linear regression model was adjusted by the following covariables; sex, age, physical activity, household size, food security, wealth index, ethnicity, education of the head, area and region. The complex sampling survey design was taken into account in all multivariate regression models. § In men≥90 cm, in women≥80 cm. ǀ The rural category included suburban population centers close to small cities, towns in rural areas distant from small towns, and disperses or very distant from rural towns.

Table 3S Adjusted difference in the ratio between energy/day intake (kilocalories) and the requirement. Overall excess weight and non-overall excess weight subjects. Colombian population, 2015.

Variable	Overall excess weight*				Non-overall excess weight				Adjusted difference‡	*P* Interaction
	n†	Mean	SE	*P* value	n†	Mean	SE	*P* value		
Sex				0.503				0.499		
Male	4062	1.03	0.02		10240	1.02	0.02		-0.03 (-0.05, 0.03)	0.794
Female	4353	1.06	0.05		10604	1.04	0.03		0.02 (-0.03, 0.07)	0.377
Age (years)				<0.0001				<0.0001		
1-4	1780	1.51	0.14		4514	1.44	0.10		0.09 (-0,01, 0.18)	0.080
5-12	1751	1.17	0.04		4424	1.17	0.03		0.00 (-0.05, 0.05)	0.955
13-17	1942	1.05	0.05		5026	1.05	0.04		-0.00 (-0.05, 0.04)	0.873
18-26	574	1.04	0.03		1413	0.99	0.03		0.03 (-0.04, 0.11)	0.390
27-49	1483	0.92	0.02		1462	0.92	0.02		0.00 (-0.04, 0.05)	0.833
50-64	750	0.85	0.02		1677	0.87	0.02		-0.02 (-0.07, 0.03)	0.379
Abdominal obesity§				0.528				0.694		
Yes	4358	1.05	0.03		550	1.04	0.04		0.00 (-0.06, 0.07)	0.880
No	957	1.07	0.06		3372	1.03	0.02		0.03 (-0.05, 0.12)	0.426
Physical activity				0.681				0.839		
Fails	5826	1.05	0.03		11229	1.03	0.02		0.01 (-0.02, 0.04)	0.976
Complies	2052	1.04	0.02		3950	1.03	0.02		-0.00 (-0.05, 0.04)	0.896
Household size				0.267				0.896		
Unipersonal	286	1.04	0.04		237	1.07	0.06		-0.01 (-0.14, 0.12)	0.869
2-4	4919	1.03	0.02		10227	1.03	0.02		-0.01 (-0.04, 0.03)	0.705
5-6	2169	1.07	0.04		6860	1.03	0.02		0.03 (-0.02, 0.07)	0.210
7+	1041	1.05	0.05		3520	1.02	0.03		0.04 (-0.03, 0.11)	0.283
Food security in the home				0.279				0.349		
Food secure	3250	1.06	0.03		6744	1.05	0.02		0.01 (-0.04, 0.05)	0.733
Mild food insecurity	2980	1.03	0.02		7455	1.02	0.02		0.01 (-0.02, 0.04)	0.574
Moderate food insecurity	1426	1.03	0.05		3891	1.01	0.03		0.01 (-0.06, 0.08)	0.767
Severe food insecurity	759	1.04	0.03		2750	1.03	0.03		0.00 (-0.06, 0.06)	0.966
Wealth index quintile				0.116				0.042		
1- poorest	4065	1.09	0.06		10586	1.06	0.04		0.03 (-0.02, 0.07)	0.286
2	2117	1.03	0.03		4989	1.05	0.03		-0.03 (-0.08, 0.01)	0.152
3	1418	1.02	0.03		3416	1.00	0.03		0.01 (-0.03, 0.06)	0.683
4- wealthiest	815	0.99	0.04		1853	0.96	0.03		0.03 (-0.05, 0.12)	0.480
Ethnicity				0.005				0.372		
Black/Afro	692	0.96	0.02		1871	1.02	0.03		-0.07 (-0.14, -0.01)	0.026
Indigenous	683	0.89	0.04		2083	0.94	0.05		-0.10 (-0.19, -0,01)	0.028
Mestizo	7011	1.06	0.03		16827	1.03	0.02		0.02 (-0.01, 0.05)	0.138
Education of the head				0.589				0.563		
Preschool or less	2119	1.02	0.02		6052	1.05	0.02		-0.03 (-0.07, 0.01)	0.191
Primary	2950	1.05	0.02		7062	1.01	0.02		0.03 (-0.01, 0.07)	0.183
Secondary	2750	1.05	0.04		6584	1.02	0.02		0.02 (-0.03, 0.07)	0.403
Post-secondary	563	1.04	0.04		995	1.05	0.03		-0.04 (-0.14, 0.06)	0.406
Area				0.929				0.785		
Urban area	6423	1.04	0.03		15494	1.03	0.02		0.01 (-0.02 0.04)	0.599
Rural‖	1992	1.05	0.03		5350	1.02	0.02		0.01 (-0.04, 0.06)	0.642
Region				0.179				0.017		
Central	2063	1.03	0.02		4956	1.05	0.01		-0.03 (-0.06, 0.01)	0.162
Atlantic	1481	1.14	0.11		4017	1.09	0.06		0.04 (-0.07, 0.15)	0.440
Oriental	1492	1.01	0.02		3672	1.00	0.02		0.02 (-0.02, 0.07)	0.365
Pacific	1058	0.99	0.04		2813	1.00	0.04		-0.01 (-0.07, 0.04)	0.607
Bogotá	568	1.02	0.04		1485	0.97	0.04		0.05 (-0.03, 0.13)	0.185
National territories	1753	1.00	0.03		3901	1.03	0.05		-0.05 (-0.12, 0.02)	0.165

* **Overall excess weight** In children and adolescents based on Z> +1, in adults≥ 25 (Kg/m^2) † In Overall excess weight *n* may be less than 8415 for missing values. In Non-overall excess weight *n* may be less than 20844 for missing values. ‡ Adjusted difference and 95% confidence interval achieved in a linear regression model with the consumption/day of kilocalories as a dependent variable and predictors that include indicator variables for each sociodemographic correlates, Non-overall excess weight (Overall excess weight) and cross-product (interaction) terms between overweight and indicator variables of the correlate. In addition, the linear regression model was adjusted by the following covariables; sex, age, physical activity, household size, food security, wealth index, ethnicity, education of the head, area and region. The complex sampling survey design was taken into account in all multivariate regression models. § In men≥90 cm, in women≥80 cm. ‖ The rural category included suburban population centers close to small cities, towns in rural areas distant from small towns, and disperses or very distant from rural towns.

Table 4S Adjusted difference in the ratio between energy/day intake (kilocalories) and the requirement. Conservative overweight and non-conservative overweight subjects. Colombian population, 2015.

Variable	Conservative overweight *				Non-conservative overweight				Adjusted difference‡	*P* Interaction
	n†	Mean	SE	*P* value	n†	Mean	SE	*P* value		
Sex				0.543				0.482		
Male	1264	1.02	0.02		13038	1.02	0.01		-0.02 (-0.06, 0.03)	0.521
Female	1367	1.06	0.06		13590	1.04	0.03		0.02 (-0.04, 0.08)	0.499
Age (years)				<0.0001				<0.0001		
1-4	568	1.55	0.16		5726	1.45	0.11		0.09 (-0.02, 0.20)	0.093
5-12	550	1.15	0.04		5625	1.18	1.11		-0.03 (-0.07, 0.01)	0.164
13-17	602	0.99	0.05		6366	1.05	0.97		-0.05 (-0.11, 0.01)	0.082
18-26	185	1.04	0.05		1802	1.00	0.95		0.02 (-0.09, 0.13)	0.674
27-49	438	0.91	0.03		4507	0.92	0.89		-0.01 (-0.07, 0.05)	0.642
50-64	247	0.89	0.04		2180	0.86	0.83		0.03 (-0.06, 0.13)	0.484
Abdominal obesity§				0.761				0.600		
Yes	1808	1.04	0.03		3100	1.05	0.03		-0.00 (-0.05, 0.04)	0.878
No	31	1.01	0.13		4298	1.04	0.03		-0.06 (-0.29, 0.17)	0.601
Physical activity				0.755				0.987		
Fails	1936	1.05	0.04		15119	1.04	0.02		0.01 (-0.03, 0.04)	0.796
Complies	587	1.04	0.03		5415	1.03	0.02		-0.00 (-0.07, 0.06)	0.894
Household size				0.031				0.871		
Unipersonal	112	0.95	0.06		411	1.08	0.04		-0.14 (-0.28, -0.00)	0.043
2-4	1603	1.02	0.03		13543	1.03	0.02		-0.01 (-0.05, 0.03)	0.510
5-6	642	1.07	0.04		8387	1.04	0.03		0.01 (-0.05, 0.08)	0.675
7+	274	1.14	0.08		4287	1.02	0.03		0.12 (-0.00, 0.24)	0.059
Food security in the home				0.923				0.209		
Food secure	1051	1.06	0.04		8943	1.05	0.02		-0.01 (-0.06, 0.04)	0.779
Mild food insecurity	981	1.02	0.03		9454	1.02	0.02		-0.00 (-0.05, 0.05)	0.962
Moderate food insecurity	374	1.03	0.07		4943	1.02	0.03		0.01 (-0.10, 0.12)	0.859
Severe food insecurity	225	1.08	0.05		3284	1.03	0.03		0.05 (-0.07, 0.16)	0.398
Wealth index quintile				0.359				0.037		
1- poorest	1303	1.09	0.07		13348	1.07	0.04		0.01 (-0.05, 0.07)	0.708
2	642	1.01	0.03		6464	1.04	0.03		-0.05 (-0.11, 0.02)	0.142
3	401	1.02	0.03		4433	1.01	0.02		-0.00 (-0.08, 0.07)	0.904
4- wealthiest	285	1.01	0.04		2383	0.96	0.02		0.05 (-0.04, 0.14)	0.293
Ethnicity				0.005				0.188		
Black/Afro	268	0.92	0.03		2295	1.01	0.03		-0.11 (-0.19, -0.02)	0.013
Indigenous	201	0.96	0.06		2565	0.93	0.05		-0.03 (-0.19, 0.13)	0.686
Mestizo	2152	1.07	0.03		21686	1.04	0.02		0.01 (-0.02, 0.05)	0.449
Education of the head				0.290				0.730		
Preschool or less	673	1.01	0.04		7498	1.04	0.02		-0.03 (-0.10, 0.03)	0.304
Primary	894	1.04	0.03		9118	1.02	0.02		0.02 (-0.04, 0.07)	0.539
Secondary	874	1.05	0.05		8460	1.03	0.03		0.01 (-0.05, 0.07)	0.828
Post-secondary	179	1.09	0.06		1379	1.04	0.03		0.04 (-0.07, 0.16)	0.431
Area				0.921				0.869		
Urban area	2037	1.04	0.04		19880	1.03	0.02		0.00 (-0.04, 0.04)	0.955
Rural‖	594	1.04	0.04		6748	1.03	0.02		0.01 (-0.07, 0.09)	0.809
Region				0.490				0.024		
Central	685	1.00	0.03		6334	1.05	0.01		-0.05 (-0.11, 0.01)	0.113
Atlantic	536	1.17	0.10		4962	1.10	0.07		0.06 (-0.02, 0.14)	0.132
Oriental	404	0.98	0.04		4760	1.01	0.02		-0.02 (-0.09, 0.06)	0.683
Pacific	339	1.01	0.04		3532	0.99	0.04		0.02 (-0.06, 0.10)	0.687
Bogotá	136	1.02	0.06		1917	0.98	0.04		0.03 (-0.09, 0.15)	0.626
National territories	531	1.01	0.04		5123	1.02	0.04		-0.02 (-0.10, 0.05)	0.544

* **Conservative overweight** In children and adolescents based on Z > +2, in adults ≥ 30 (Kg/m^2) † In conservative overweight *n* may be less than 2631 for missing values. In Non-conservative overweight *n* may be less than 26628 for missing values. ‡ Adjusted difference and 95% confidence interval achieved in a linear regression model with the consumption/day of kilocalories as a dependent variable and predictors that include indicator variables for each sociodemographic correlates, Non-conservative overweight (Conservative overweight) and cross-product (interaction) terms between obese and indicator variables of the correlate. In addition, the linear regression model was adjusted by the following covariables; sex, age, physical activity, household size, food security, wealth index, ethnicity, education of the head, area and region. The complex sampling survey design was taken into account in all multivariate regression models. § In men≥90 cm, in women≥80 cm. ‖ The rural category included suburban population centers close to small cities, towns in rural areas distant from small towns, and disperses or very distant from rural towns.

Table 5S Adjusted difference in the ratio between protein intake and adequate weight (g/kg/d). Overall excess weight and non-overall excess weight subjects. Colombian population, 2015.

Variable	Overall excess weight*				Non-overall excess weight				Adjusted difference‡	*P* Interaction
	n†	Mean	SE	*P* value	n†	Mean	SE	*P* value		
Sex				0.437				0.362		
Male	4062	1.72	0.06		10240	1.67	0.05		0.03 (-0.06, 0.11)	0.554
Female	4353	1.61	0.13		10604	1.58	0.08		0.03 (-0.10, 0.16)	0.692
Age (years)				<0.0001				<0.0001		
1-4	1780	3.94	0.25		4514	3.81	0.17		0.12 (-0.09, 0.33)	0.269
5-12	1751	2.24	0.07		4424	2.25	0.07		-0.01 (-0.10, 0.07)	0.745
13-17	1942	1.54	0.12		5026	1.53	0.10		-0.02 (-0.11, 0.06)	0.615
18-26	574	1.25	0.04		1413	1.27	0.03		-0.04 (-0.14, 0.05)	0.343
27-49	1483	1.12	0.02		1462	1.13	0.03		0.00 (-0.07, 0.07)	0.957
50-64	750	1.10	0.04		1677	1.03	0.03		0.03 (-0.06, 0.12)	0.469
Abdominal obesity§				0.265				0.311		
Yes	4358	1.64	0.08		550	1.69	0.10		-0.03 (-0.18, 0.12)	0.710
No	957	1.72	0.12		3372	1.61	0.06		0.08 (-0.07, 0.22)	0.590
Physical activity				0.758				0.641		
Fails	5826	1.67	0.09		11229	1,64	0.05		0.03 (-0.04, 0.11)	0.360
Complies	2052	1.64	0.06		3950	1.65	0.05		0.01 (-0.09, 0.10)	0.850
Household size				0.945				0.715		
Unipersonal	286	1.63	0.10		237	1.75	0.12		-0.08 (-0.38, 0.21)	0.579
2-4	4919	1.65	0.06		10227	1.62	0.04		0.01 (-0.06, 0.08)	0.785
5-6	2169	1.72	0.09		6860	1.67	0.06		0.06 (-0.04, 0.16)	0.214
7+	1041	1.59	0.14		3520	1.57	0.09		0.05 (-0.12, 0.23)	0.550
Food security in the home				0.026				0.082		
Food secure	3250	1.71	0.09		6744	1.68	0.05		0.01 (-0.09, 0.11)	0.874
Mild food insecurity	2980	1.65	0.06		7455	1.62	0.06		0.04 (-0.04, 0.12)	0.287
Moderate food insecurity	1426	1.60	0.11		3891	1.54	0.06		0.07 (-0.07, 0.21)	0.344
Severe food insecurity	759	1.58	0.08		2750	1.63	0.07		-0.06 (-0.19, 0.08)	0.425
Wealth index quintile				0.534				0.579		
1- poorest	4065	1.74	0.17		10586	1.66	0.11		0.07 (-0.06, 0.20)	0.266
2	2117	1.61	0.07		4989	1.65	0.06		-0.06 (-0.15, 0.03)	0.217
3	1418	1.58	0.07		3416	1.57	0.07		-0.01 (-0.13, 0.10)	0.805
4- wealthiest	815	1.66	0.08		1853	1.61	0.07		0.08 (-0.08, 0.25)	0.318
Ethnicity				0.003				0.032		
Black/Afro	692	1.40	0.07		1871	1.47	0.08		-0.11 (-0.26, 0.04)	0.161
Indigenous	683	1.29	0.07		2083	1.46	0.08		-0.25 (-0.43, -0.08)	0.006
Mestizo	7011	1.63	0.08		16827	1.65	0.06		0.05 (-0.02, 0.12)	0.140
Education of the head				0.107				0.131		
Preschool or less	2119	1.54	0.06		6052	1.58	0.06		-0.02 (-0.10, 0.06)	0.665
Primary	2950	1.69	0.06		7062	1.64	0.05		0.02 (-0.06, 0.09)	0.659
Secondary	2750	1.70	0.11		6584	1.65	0.07		0.06 (-0.05, 0.17)	0.277
Post-secondary	563	1.72	0.10		995	1.70	0.07		-0.00 (-0.23, 0.22)	0.971
Area				0.015				0.010		
Urban area	6423	1.71	0.09		15494	1.68	0.06		0.04 (-0.05, 0.12)	0.383
Rural‖	1992	1.47	0.04		5350	1.47	0.05		0.00 (-0.09, 0.09)	0.999
Region				0.948				0.964		
Central	2063	1.60	0.10		4956	1.62	0.08		-0.02 (-0.10, 0.06)	0.603
Atlantic	1481	1.84	0.29		4017	1.67	0.17		0.16 (-0.11, 0.44)	0.240
Oriental	1492	1.59	0.04		3672	1.63	0.04		-0.01 (-0.11, 0.08)	0.767
Pacific	1058	1.44	0.10		2813	1.48	0.09		-0.06 (-0.17, 0.05)	0.270
Bogotá	568	1.72	0.10		1485	1.68	0.09		0.06 (-0.11, 0.23)	0.484
National territories	1753	1.81	0.10		3901	1.76	0.15		0.04 (-0.15, 0.24)	0.675

* **Overall excess weight** In children and adolescents based on Z> +1, in adults≥ 25 (Kg/m^2) † In Overall excess weight *n* may be less than 8415 for missing values. In Non-overall excess weight *n* may be less than 20844 for missing values. ‡ Adjusted difference and 95% confidence interval achieved in a linear regression model with the consumption/day of kilocalories as a dependent variable and predictors that include indicator variables for each sociodemographic correlates, Non-overall excess weight (Overall excess weight) and cross-product (interaction) terms between overweight and indicator variables of the correlate. In addition, the linear regression model was adjusted by the following covariables; sex, age, physical activity, household size, food security, wealth index, ethnicity, education of the head, area and region. The complex sampling survey design was taken into account in all multivariate regression models. § In men≥90 cm, in women≥80 cm. ‖ The rural category included suburban population centers close to small cities, towns in rural areas distant from small towns, and disperses or very distant from rural towns.

Table 6S Adjusted difference in the ratio between protein intake and the adequate weight (g/kg/d). Conservative overweight and non-conser overweight subjects. Colombian population, 2015.

	Conservative overweight [*]				Non-conservative overweight				Adjusted	*P*
Variable	n[†]	Mean	SE	*P* value	n[†]	Mean	SE	*P* value	difference[‡]	Interaction
Sex				0.756				0.340		
Male	1264	1.70	0.07		13038	1.68	0.05		-0.01 (-0.11, 0.09)	0.814
Female	1367	1.64	0.16		13590	1.59	0.09		0.06 (-0.10, 0.22)	0.483
Age (years)				<0.0001				<0.0001		
1-4	568	3.91	0.30		5726	3.84	0.18		0.04 (-0.22, 0.31)	0.738
5-12	550	2.27	0.09		5625	2.25	0.07		0.00 (-0.10, 0.11)	0.979
13-17	602	1.45	0.11		6366	1.54	0.10		-0.09 (-0.20, 0.01)	0.085
18-26	185	1.17	0.06		1802	1.27	0.03		-0.13 (-0.27, 0.02)	0.079
27-49	438	1.10	0.04		4507	1.13	0.02		-0.02 (-0.11, 0.07)	0.666
50-64	247	1.15	0.07		2180	1.03	0.03		0.13 (-0.02, 0.29)	0.097
Abdominal obesity[d]				0.599				0.618		
Yes	1808	1.64	0.09		3100	1.65	0.07		0.00 (-0.10, 0.11)	0.935
No	31	1.78	0.30		4298	1.63	0.07		0.06 (-0.42, 0.54)	0.791
Physical activity				0.940				0.878		
Fails	1936	1.67	0.10		15119	1.65	0.06		0.02 (-0.07, 0.11)	0.618
Complies	587	1.68	0.10		5415	1.65	0.05		0.03 (-0.11, 0.17)	0.695
Household size				0.187				0.516		
Unipersonal	112	1.54	0.14		411	1.73	0.09		-0.19 (-0.52, 0.13)	0.244
2-4	1603	1.62	0.08		13543	1.63	0.05		-0.02 (-0.11, 0.07)	0.627
5-6	642	1.75	0.11		8387	1.68	0.06		0.07 (-0.07, 0.20)	0.337
7+	274	1.79	0.22		4287	1.56	0.09		0.26 (-0.05, 0.58)	0.101
Food security in the home				0.613				0.026		
Food secure	1051	1.71	0.12		8943	1.69	0.05		-0.02 (-0.15, 0.12)	0.791
Mild food insecurity	981	1.63	0.07		9454	1.63	0.06		0.02 (-0.09, 0.12)	0.782
Moderate food insecurity	374	1.61	0.17		4943	1.55	0.07		0.09 (-0.15, 0.33)	0.453
Severe food insecurity	225	1.71	0.12		3284	1.61	0.07		0.12 (-0.11, 0.35)	0.312
Wealth index quintile				0.599				0.551		
1- poorest	1303	1.77	0.21		13348	1.68	0.12		0.06 (-0.08, 0.20)	0.374
2	642	1.54	0.07		6464	1.65	0.06		-0.12 (-0.25, 0.00)	0.057
3	401	1.64	0.08		4433	1.57	0.07		0.04 (-0.11, 0.20)	0.574
4- wealthiest	285	1.64	0.10		2383	1.62	0.07		0.06 (-0.14, 0.26)	0.551
Ethnicity				0.027				0.012		
Black/Afro	268	1.41	0.09		2295	1.45	0.07		-0.07 (-0.27, 013)	0.496
Indigenous	201	1.50	0.13		2565	1.42	0.07		-0.07 (-0.39, 0.24)	0.643
Mestizo	2152	1.70	0.10		21686	1.66	0.06		0.04 (-0.06, 0.13)	0.449
Education of the head				0.046				0.116		
Preschool or less	673	1.50	0.08		7498	1.57	0.05		-0.05 (-0.19, 0.09)	0.467
Primary	894	1.69	0.07		9118	1.65	0.05		0.02 (-0.08, 0.13)	0.657
Secondary	874	1.72	0.15		8460	1.66	0.07		0.05 (-0.09, 0.19)	0.493
Post-secondary	179	1.85	0.13		1379	1.69	0.06		0.14 (-0.12, 0.41)	0.278
Area				0.127				0.007		
Urban area	2037	1.70	0.11		19880	1.66	0.06		0.01 (-0.09, 0.11)	0.865
Rural[ǀ]	594	1.52	0.06		6748	1.47	0.04		0.09 (-0.04, 0.21)	0.172
Region				0.812				0.973		
Central	685	1.59	0.12		6334	1.62	0.09		-0.04 (-0.16, 0.08)	0.491
Atlantic	536	1.88	0.30		4962	1.70	0.19		0.15 (-0.07, 0.36)	0.171
Oriental	404	1.53	0.08		4760	1.63	0.04		-0.08 (-0.26, 0.09)	0.344
Pacific	339	1.45	0.10		3532	1.47	0.09		-0.02 (-0.18, 0.13)	0.749
Bogotá	136	1.86	0.15		1917	1.69	0.09		0.15 (-0.12, 0.43)	0.272
National territories	531	1.83	0.12		5123	1.77	0.14		0.05 (-0.21, 0.30)	0.722

[*] **Conservative overweight** In children and adolescents based on Z > +2, in adults ≥ 30 (Kg/m^2) [†] In conservative overweight *n* may be less than 2631 for missing values. In Non-conservative overweight *n* may be less than 26628 for missing values. [‡] Adjusted difference and 95% confidence interval achieved in a linear regression model with the consumption/day of kilocalories as a dependent variable and predictors that include indicator variables for each sociodemographic correlates, Non-conservative overweight (Conservative overweight) and cross-product (interaction) terms between obese and indicator variables of the correlate. In addition, the linear regression model was adjusted by the following covariables; sex, age, physical activity, household size, food security, wealth index, ethnicity, education of the head, area and region. The complex sampling survey design was taken into account in all multivariate regression models. [§] In men≥90 cm, in women≥80 cm. [ǀ] The rural category included suburban population centers close to small cities, towns in rural areas distant from small towns, and disperses or very distant from rural towns.
